# Carotid duplex parameters to predict long term outcomes of ischemic stroke patients receiving intra-arterial thrombectomy treatment

**DOI:** 10.1097/MD.0000000000015734

**Published:** 2019-05-17

**Authors:** Yu-Jun Chang, Chih-Ming Lin, Yang-Hao Ou, Chi-Kuang Liu, Wei-Liang Chen, Shih-Liang Chang

**Affiliations:** a Epidemiology and Biostatistics Center; b Department of Neurology, Changhua Christian Hospital, Changhua; c Department of Social Work and Child Welfare, Providence University, Taichung; d Department of Medicinal Botanicals and Health Applications, Da-Yeh University; e Department of Medical Imaging, Changhua Christian Hospital, Changhua; f School of Chinese Medicine, China Medical University, Taichung, Taiwan.

**Keywords:** intra-arterial thrombectomy, intravenous thrombolysis, ischemic stroke, pulsatility index, resistance index

## Abstract

Early treatment of acute ischemic stroke with intravenous thrombolysis therapy (ITT) followed by intra-arterial thrombectomy (IAT) is a promising new treatment option for improving functional outcomes. Identifying patients who will benefit from this treatment combination is important.

A total of 92 acute ischemic stroke patients who received ITT and IAT with a minimum of 1-year follow-up were included in the study. All parameters of clinical and imaging examinations at baseline were examined which parameters were significantly correlated with the 1-year functional outcomes (modified Rankin scale [mRS], National Institute of Health Stroke Scale [NIHSS], and Barthel Index) after stroke. Receiver-operating characteristic (ROC) curves analysis was performed to estimate the diagnostic performance of each significantly related parameter. Youden index was used to determine the optimal threshold value. Multivariate logistic regression model analyses were applied to verify the results of predicting the favorable functional outcomes.

Immediate postoperation outcome with modified thrombolysis in cerebral infarction grading showed that total of 62 patients qualified for satisfactory result (2b or 3). In predicting NIHSS improvement, ROC curve analysis showed that a cutoff point of vertebral artery pulsatility index (VA PI)-ipsilateral ≤2.3 yields the best diagnostic performance (area under the ROC curve [AUC] = 0.728); in predicting mRS improvement, VA PI-ipsilateral ≤1.92 and internal carotid artery resistance index (ICA RI)-ipsilateral ≤0.71 yield good diagnostic performance (AUC = 0.697 and 0.672, respectively); and ICA RI-contralateral ≤0.70 or plaque index-ipsilateral ≤2 had better diagnostic accuracy (AUC = 0.764 and 0.689, respectively) than other indices to predict Barthel index improvement. The multivariate analysis also showed that these 5 indices were those more powerful and highly significant favorable functional outcomes predictors.

Parameters of pulsatility and resistance index from carotid duplex could be easily accessed and noninvasive. The outcome of ischemic stroke patients receiving ITT followed by IAT can be forecasted by these 2 crucial predictors that hint the patients’ functional outcomes as well as guiding first line in-charge clinician in terms of decision making.

## Abstract 2

Supplemental Digital Content is available in the text

## 1 Introduction

Timely treatment of acute ischemic stroke (AIS) with intravenous thrombolysis therapy (ITT) with recombinant tissue plasminogen activator (alteplase or rtPA) has long been the gold standard worldwide.^[1]^ Recently, multiple randomized control trials studied the outcome of ITT followed by intra-arterial thrombectomy (IAT) and demonstrated a significant increase in rates of revascularization in subsets of patients.^[2–5]^ The 2018 American Heart and Stroke Associations’ (AHA/ASA) guidelines have recommended this treatment method in appropriately selected patients.^[6]^ However, to date, there is limited data on such intervention methods, largely due to the high degree of heterogeneity in data and mixed results in these studies.^[7]^ Therefore, further research is required to determine which factors may help identify patients who are suitable for this emerging treatment method.^[8]^

The 2016 meta-analysis by Noguiera et al suggested that cerebral circulation parameters obtained pre-, during, and postintervention were useful in predicting a patient's clinical outcome after thrombolysis. It was observed that patients with tandem internal carotid artery (ICA) or middle cerebral artery (MCA) occlusion alone had poorer functional outcomes than those with isolated MCA occlusion.^[9]^ This suggested it is of critical importance to determine the hemodynamic status of the large vessels (common carotid artery [CCA], ICA, and vertebral artery (VA)) in predicting a patient's outcome.

Carotid doppler is a simple, accurate, and reproducible method which measures peak systolic velocity (PSV), end diastolic velocity (EDV), mean flow velocity and peak diastolic velocity. These parameters can be used to calculate the resistive index (RI) and pulsatility index (PI), which have been shown to be the most practical parameters for assessing the hemodynamic status of CCA, ICA, extracranial carotid artery (ECA), and ophthalmic artery(OA).^[10]^ Additionally, these same parameters have also been identified as potentially useful markers of functional outcome following carotid artery stenting procedures. In a study of 67 patients who underwent stenting procedures following ischemic stroke, it was shown that the net contralateral ICA resistance index (RI) was significantly lower for patients who had improved functional outcome, assessed by the modified Ranking scale (mRS).^[11]^

The primary goal for this study was to investigate whether doppler or other imaging parameters, especially RI and PI measured by carotid duplex exam, could predict functional outcomes (as assessed by the National Institute of Health Stroke Scale [NIHSS], mRS, and Barthel index) in ischemic stroke patients who had undergone ITT followed by IAT. The secondary aim was to determine whether cut-off points exist that could provide important clinical information regarding stroke patients’ long term outcomes. We hypothesize that these parameters play an important role in selecting suitable patients for IAT and can be used for early prediction of long-term outcomes.

## 2 Materials and methods

### 2.1 Study design

This was a single center, retrospective medical record review conducted at the angiography laboratory of the Department of Neuroimaging, Changhua Christian Hospital, Taiwan. Patients were selected who had been diagnosed with AIS and had undergone ITT followed by IAT using standardized protocols (the ITT window was set at ≦4.5 hours and IAT ≦8 hours from symptom onset; the detailed protocol information was listed in the intravenous thrombolytic therapy and angiography/IAT section). Three different outcome measurements (the NIHSS score, mRS score, and Barthel index) were compared at 2-time points: preintervention and at 12 months postintervention. Data extraction was conducted by 2 independent clinicians. The study protocol was approved by the Changhua Christian Hospital ethics committee.

### 2.2 Patient selection

Data were obtained from 92 AIS patients, confirmed by neuroimaging, who were treated with both ITT and IAT between January 2015 and December 2017. All patients included in the study had anterior circulation stenosis and/or obstruction that met the predefined inclusion and exclusion criteria. Patients were selected who were ≥18 years of age and were followed up for at least 12 months. The exclusion criteria were patients with intracerebral hemorrhage, cerebral arteriovenous malformations or aneurysms, or recurrent stroke during the period of study.

### 2.3 Cervical carotid ultrasound examination

All procedures were conducted by a neurointerventional team in a specialized angiography clinic at the Changhua Christian Hospital, Taiwan. Cervical carotid artery ultrasound examination (Philips iE33 7-Mhz linear transducer) was performed upon the patient once they arrived at the emergency room. Cross-sectional B-mode scanning was performed to check for intraluminal plaque material and the longitudinal screening method was adopted to confirm the presence of plaque. Two physicians assessed and classified plaques into subtypes 1, 2, 3, or 4, according to the International Classification System.^[12]^ In cases where there was disagreement between the physicians, a third physician made the final assessment.

The intima-media thickness of the mid-portion of the CCA was measured on the ipsilateral side of the index stroke event. The parameters of PSV, EDV, RI (calculated as [PSV-EDV]/PSV) and PI (calculated as [PSV-EDV]/mean of the velocity) of the CCA, ICA, ECA, VA, and OA were measured bilaterally. The reversal of blood flow in the OA was also measured. Forward flow was defined as blood flow detected out of the stenotic ipsilateral carotid artery, whereas reverse flow was defined as blood flow into the carotid artery. The machine automatically calculated the plaque index^[13]^ on both the ipsilateral and contralateral sides of the cerebral lesion. The approximate time of assessing the extra-cranial artery blood flows by carotid duplex was 30 minutes. It is to evaluate patients having major carotid artery severe stenosis/obstruction exited

### 2.4 Angiography/IAT

The indications for IAT were findings of major artery stenosis (visualized and confirmed by a neuroradiologist) with a suitable access location in order for the procedure to be carried out, that is, terminal intracranial ICA, and the first or second branch of the MCA. The IAT procedure was performed with informed consent from the patient or family. One 8F right femoral sheath was inserted and 1 6F Neuro Max long sheath was advanced up to the stenotic site, then 1 3Max Penumbra aspiration reperfusion catheter was inserted coaxially via the guiding catheter and navigated to the proximal part of the occluded MCA to remove the thrombus.

A post-procedural immediate outcome was evaluated by a neuroradiologist using the modified thrombolysis in cerebral infarction (mTICI) score.^[14,15]^ The TICI score was defined as the following: 0 = no perfusion; 1 = penetration, but no distal branch filling; 2a = perfusion with incomplete (<50%) distal branch filling; 2b = perfusion with incomplete (>50%) distal branch filling; and 3 = full reperfusion with filling of all distal branches. A satisfactory result was defined as a TICI score of 2b or 3.

### 2.5 Cerebral CT angiography (CTA) and CT perfusion (CTP) protocol

The computed tomography (CT) stroke protocol was performed on a dual source CT scanner (Siemens Definition Flash). Pre- and post-contrast CT scans of the head with the following parameters were performed: 120 kilovolts (peak) (kVp), 220 mA (auto), 64 × 0.6 mm collimation, 0.28 s/rotation, and table speed of 1 mm/rotation; CT angiography was performed from the aortic arch to the vertex with the following parameters: total 60cc of iodinated contrast agent was injected at 5 ml/s (Iohexol, Omnipaque, 350 mg iodine/ml; GE Healthcare, Piscataway, NJ), 5- to 10-second delay, 100/140 kVp, auto mA, 0.28 s/rotation, 0.6-mm-thick sections, and table speed of 4 cm/rotation. CTA data were automatically processed by the technicians, including multiplanar 5 mm maximum intensity projection reconstructions and 5-mm axial reformates or CTA source images.

The CTP technique included 45-second scanning reconstructed at 0.5-second intervals to produce a series of 90 sequential images for each of the 8 sections, covering a total of 40 mm from the basal ganglia to the lateral ventricles. CTP scanning parameters were the following: 80 kVp, 150 mA, total 50cc iodinated contrast agent injected at the rate of 5 ml/s.

### 2.6 Intravenous thrombolytic therapy

All patients received pre-interventional ITT with rtPA as bridging therapy for IAT. (The time window for ITT was set at ≦4.5 hours from symptom onset) A thorough patient evaluation including neuroimaging was carried out before the administration of r-tPA, and the patient's stroke severity was scored using the NIHSS score. Shortly after r-tPA ITT therapy, suitability for IAT was evaluated using perfusion scanning CTA/P. The allowed time window for IAT to the anterior circulatory system was set at ≦ 8 hours after the onset of symptoms. Before commencing the IAT, patient baseline characteristics were documented, including demographic information, NIHSS score, Barthel index, mRS, blood biochemistry, carotid duplex, and CTA/P.

### 2.7 Angiography and IAT protocol

Under general endotracheal anesthesia, 1 9F right femoral sheath was inserted through the right femoral artery and then a Neuron Max 088 catheter (Penumbra Inc, Alameda, CA) with a coaxial JB2 catheter (Cook Medical Inc, Bloomington, IN) was advanced to the CCA or ICA. A diagnostic cerebral angiogram was performed to confirm the location and extension of the blood clot. Mechanical thrombectomy was initiated using the Penumbra aspiration catheter (Penumbra Inc) or Solitaire Platinum revascularization device (Medtronics Inc, Minneapolis, MN).

### 2.8 Outcome measurements

Outcome measurement parameters included the NIHSS score, Barthel index, and mRS score. These parameters were re-evaluated 1 year after the rtPA and IAT bridging therapy, which was conducted in an outpatient clinic setting.

### 2.9 Statistical analysis

The baseline clinical and imaging examination data were used to correlate 3 1-year functional outcomes (as assessed using the NIHSS score, mRS score, and Barthel index) after stroke. Each functional outcome was divided into 2 groups: improvement and no improvement. Statistical tests used included Student *t* test or Mann–Whitney *U* test (for continuous variables) and Chi-square test or Fisher exact test (for categorical variables), as appropriate. Receiver operating characteristic (ROC) curve was used to identify the variable with the best diagnostic power. The Youden index was used to determine the optimal cut-off value. Pearson correlation analysis was performed to identify the correlation between all the measured indicators and to confirm the results of PI and RI measurements for each artery were consistent. Once potentially predictors were identified, a multiple logistic regression model simultaneously determined the most important prognostic variables and estimates their correlation strength. The final model only retained the significant predictors (*P* < .05). All statistical analyses were performed using the IBM SPSS Statistics for Windows, Version 22.0 (IBM Corp, Armonk, NY).

## 3 Results

### 3.1 Baseline characteristics

A total of 92 patients who were treated between January 2015 and December 2017 and had a minimum of 1-year follow-up data were included in the study. Baseline demographic characteristics, assessment (clinical, radiographic, and sonographic) measures, and 3 primary outcomes (NIHSS, mRS, and Barthel index) were described in Tables 1 and 2. The mean age of the participants was 65 years old (SD = 15), of which 53 participants (57.6%) were men. There were 42 patients (45.7%) in the ICA and 71 patients (77.2%) in the VA with proximal arterial occlusion or stenosis (RI >= 0.75). The pre-intervention mean NIHSS score was 20.98 ± 7.32, the mean mRS score was 4.54 ± 0.69, and the mean Barthel index was 13.42 ± 13.98.

**Table 1:**
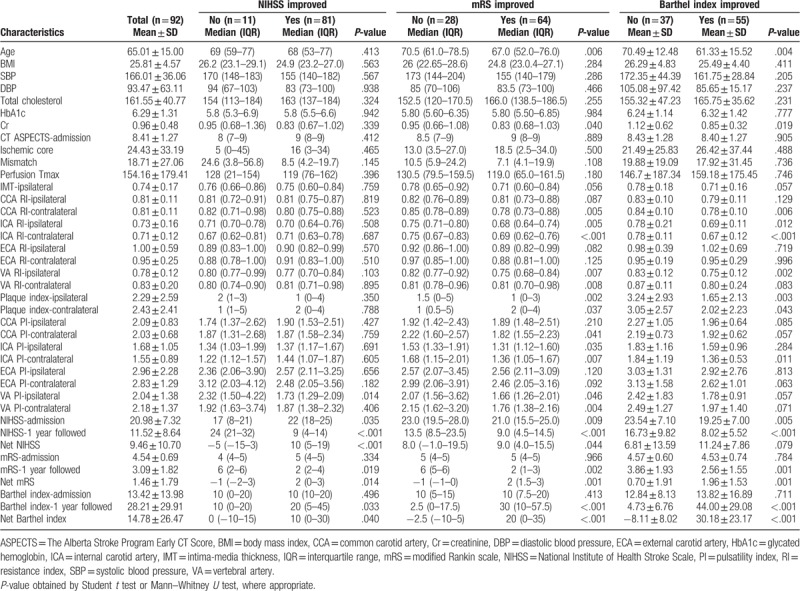
Baseline demographic, clinical characteristics, and 3 outcome measurements.

**Table 2:**
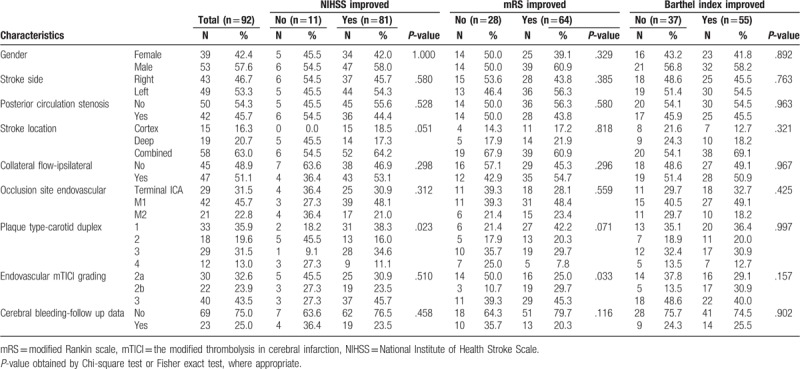
The features of 3 outcome measurements in pretreatment stage.

### 3.2 Intervention details

Out of 92 patients who underwent ITT followed by IAT for AIS of large vessels, 17 (18.5%) patients had experienced at least 1 prior stroke. In total, 29 patients had terminal ICA occlusion, 42 patients had MCA M1 segment occlusion, and 21 had M2 segment occlusion. The immediate postoperation outcome as determined by TICI grading showed that a total of 62 patients qualified for satisfactory result (TICI grade of 2b or 3), demonstrating a successful outcome in 67% of patients. The remaining 30 patients received partial recanalization, or a TICI grade of 2a or less (Table 2).

### 3.3 Primary outcomes and potential predictors

The primary outcome analysis showed there was a net improvement of all 3 scores at 1-year postintervention (Table 1). The NIHSS score was decreased by a net amount of 9.46 ± 10.70, the mRS score showed a net decrease of 1.46 ± 1.79, and a net increase of 14.78 ± 26.47 in Barthel index was observed.

To understand which parameters are associated with the 3 primary outcomes, all patients were further broken down into the improved or nonimproved groups in each of the 3 outcome scores for each of the parameters that were measured. For the NIHSS score, only the VA ipsilateral PI parameters were significantly different between the improved and unimproved groups (*P* = .014). In terms of mRS scores, ipsilateral RI parameters for both ICA and VA were significantly different between the improved and unimproved groups (*P* = .005 and .007, respectively). For the Barthel index, the most significant differences were in the ICA contralateral RI (*P* < .001), VA ipsilateral RI (*P* = .002) and ipsilateral plaque index (*P* = .003) parameters (Table 1).

### 3.4 ROC curve analysis

**Figure 1:**
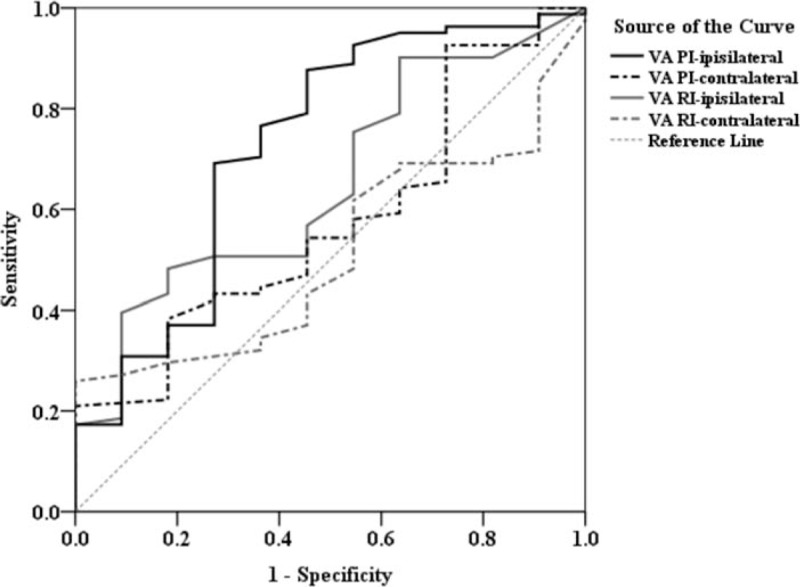
Comparison of the diagnostic power of VA PI-ipsilateral, VA PI-contralateral, VA RI-ipsilateral, and VA RI-contralateral of ROC curve analysis in predicting whether NIHSS will improve at 1 year after stroke. NIHSS = National Institute of Health Stroke Scale, ROC = receiver operating characteristic, VA PI = vertebral artery pulsatility index, VA RI = vertebral artery resistive index.

**Figure 2:**
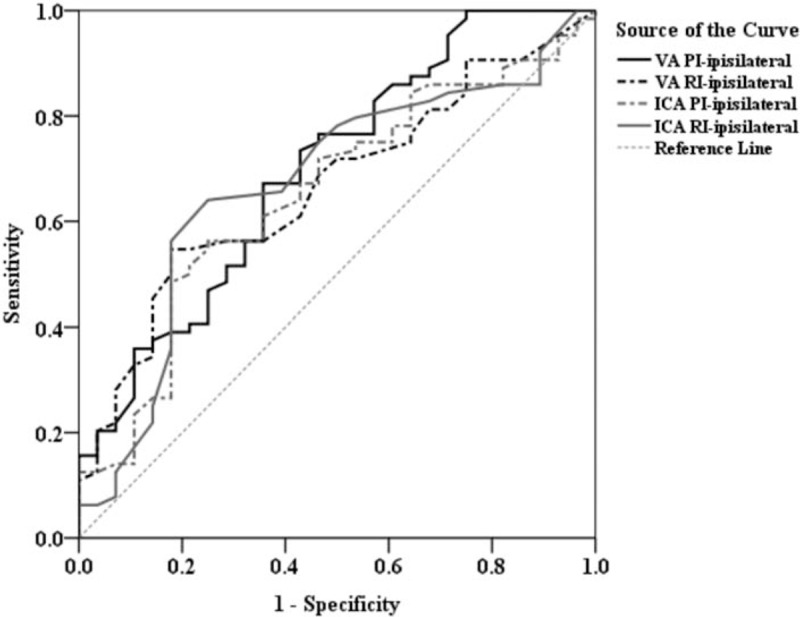
Comparison of the diagnostic power of VA PI-ipsilateral, VA RI-ipsilateral, ICA PI-contralateral, and ICA RI-contralateral of ROC curve analysis in predicting whether mRS will improve at 1 year after stroke. ICA PI = internal carotid artery pulsatility index, ICA RI = internal carotid artery resistive index, mRS = modified Ranking scale, ROC = receiver operating characteristic, VA PI = vertebral artery pulsatility index, VA RI = vertebral artery resistive index.

**Figure 3:**
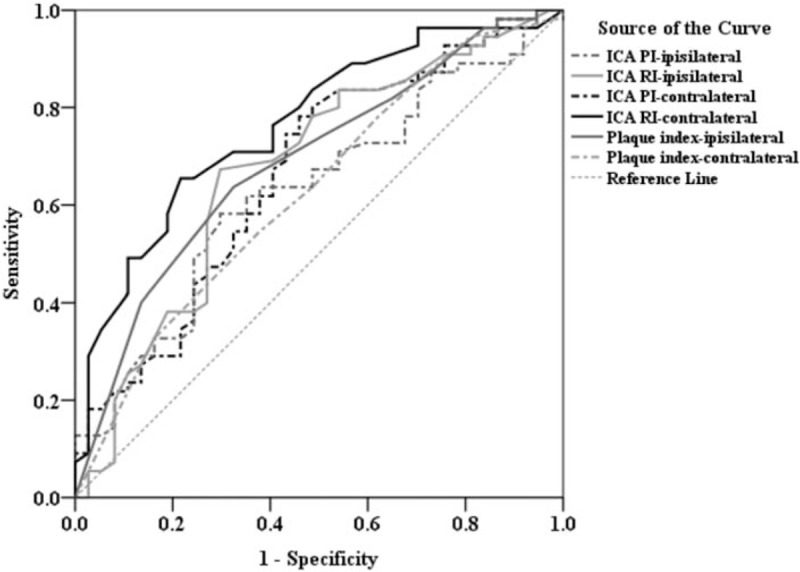
Comparison of the diagnostic power of ICA PI-ipsilateral, ICA RI-ipsilateral, ICA PI-ipsilateral, and ICA RI-ipsilateral of ROC curve analysis in predicting whether Barthel Index will improve at 1 year after stroke. ICA PI = internal carotid artery pulsatility index, ICA RI = internal carotid artery resistive index, ROC = receiver operating characteristic.

After identifying the carotid doppler parameters that were the most significant differences in the 3 outcome measurements, we used the ROC curve analysis to assess the diagnostic performance for each resistance and PI and further determined a specific cutoff point in each of these parameters which could best predict a beneficial outcome in patients who had undergone ITT followed by IAT. The area under the ROC curve (AUC), cutoff value, sensitivity, and specificity were analyzed (Figs. 1–3; summarized in Table 3). The optimal cutoff value was defined as the point that yielded the best sensitivity and specificity for the differentiation. Among the 4 VA resistance and pulsatility indices, VA PI-ipsilateral was the best index for the screening of NIHSS improvement in those patients (AUC = 0.728), and the optimal cutoff of VA PI-ipsilateral ≤2.3 has a sensitivity of 87.7%, specificity of 54.5%, positive predictive value (PPV) of 93.4%, and negative predictive value (NPV) of 37.5%. Similarly, in predicting mRS improvement, the VA PI-ipsilateral had the best diagnostic ability performance (AUC = 0.697), followed by ICA RI-ipsilateral (AUC = 0.697), VA RI-ipsilateral (AUC = 0.665), and ICA PI-ipsilateral (AUC = 0.651). Youden index established the optimal threshold values of ≤1.92 for VA PI-ipsilateral (sensitivity = 67.2%, specificity = 64.3%, PPV = 81.1%, NPV = 46.2%) and ≤ 0.70 for ICA RI-ipsilateral (sensitivity = 64.1%, specificity = 75.0%, PPV = 79.2%, NPV = 43.6%). And finally, ROC curve revealed ICA RI-contralateral and plaque index-ipsilateral had a better performance (AUC = 0.764 and 0.689, respectively) than other indices to predict Barthel index improvement. The optimal cutoff points for ICA RI-contralateral and plaque index-ipsilateral were ≤0.70 (sensitivity = 65.5%, specificity = 78.4%, PPV = 80.0%, NPV = 59.6%) and ≤2 (sensitivity = 63.6%, specificity = 67.6%, PPV = 68.3%, NPV = 56.3%), respectively.

**Table 3:**
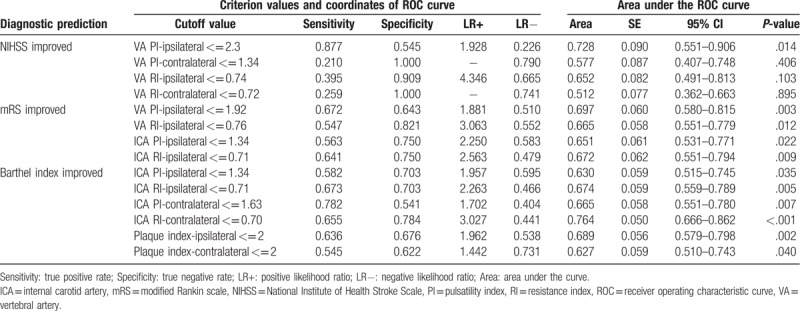
Receiver operating characteristic (ROC) curve analyses of 3 outcome measurements.

### 3.5 Multiple logistic regression analysis

To more accurately verify the diagnostic and predictive capabilities of each of these cut-off points, we performed multiple logistic regression analyses to control interference of confounding factors. Table 4 shows that when VA PI-ipsilateral values >2.3, the odds ratio (OR) of improved NIHSS was 0.102 (95% confidence interval [CI] = 0.019–0.544, *P* = .008); similarly, when VA PI-ipsilateral value >1.92, the OR of improved mRS was 0.337 (95% CI = 0.123–0.921, *P* = .034), and when ICA RI-ipsilateral >0.75, the OR of improved mRS was 0.287 (95% CI = 0.099–0.830, *P* = .021); lastly, when ICA RI-contralateral value >0.7, the OR of improved Barthel index was 0.288 (95% CI = 0.101–0.822, *P* = .02).

**Table 4:**
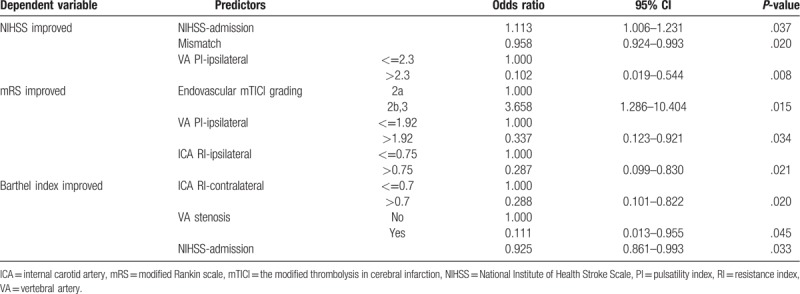
Multiple logistic regression analyses of 3 outcome measurements.

## 4 Discussion

ITT remains the gold standard and more effective treatment option for ischemic patients in terms of large artery recanalization. In recent years, ITT followed by IAT has been adopted and proven to be more efficacious than ITT alone or other oral medication, such as antiplatelet (aspirin), anticoagulants (warfarin), or new generation therapeutic agents (dabigatran). Although the current guidelines recommend ITT followed by IAT therapy, clinical practice is still in the initial stages, and the long term outcomes for patients following this treatment strategy remain unknown. There is a clinical need to identify which patients will have optimal benefit from IAT therapy. In this study, we have demonstrated the importance of PI and RI parameters obtained from carotid duplex scanning in predicting the likelihood of favorable patient outcomes after receiving the bridging therapy. Moreover, we have identified cut-off values of several parameters which could predict beneficial outcomes in individual patients.

As there were a large number of clinical characteristics measured in our study, we initially correlated all the pretreatment baseline variables with the 3 outcome measurements (shown in Table 1, Table 2, and Supplementary Table 1). Except for the carotid duplex parameters, the others seem to be less relevant. Thus we focused our study on the variables derived from the carotid duplex exam and the functional outcomes after receiving the bridging therapy.

Although the mean age of our study population was relatively old (65 years of age), the immediate success rate of patients following the ITT with IAT therapy was over 60%. The guidelines from AHA/ASA recommend that stroke patients of an advanced age (≥80 years old) are more prone to developing an intra-cerebral hemorrhage after the procedure. This is likely due to the fact that the intra-cerebral vasculature in elderly patients is more fragile compared with younger patients, and they are therefore susceptible to hemorrhagic transformation which is associated with increased mortality and morbidity. Despite this, a recent study reported that a subset of older patients do indeed benefit from ITT plus IAT therapy^[16]^; and so there is a need to identify this subset of patients in older populations as well.

A review of the current literature shows that PI is commonly used for the assessment of intracranial blood flow velocity and has been demonstrated to correlate with intracranial pressure,^[16]^ while RI measurements can aid in the evaluation of cerebral blood flow via extracranial carotid and vertebral arteries.^[17]^ The normal values for PI and RI are typically <1.2 and <0.75, respectively. However, to the best of our knowledge, any correlation between PI and RI values, or the magnitude of increase in values of PI and RI, remains unknown. In this study, we observed a positive correlation between PI and RI (Supplementary Table 2) in the CCA, ICA, and VA. Thus, we hypothesize that clinicians could use PI to predict the outcome of IAT.

One shortcoming of current adopted normal values of PI and RI is that there is only a choice between simple “yes obstruction” and “no obstruction” categories when classifying the hemodynamics of large vessels. Such classification is not practical or sufficient for both physicians and the patients to make appropriate decisions regarding the outcomes of IAT after ITT. Because of this limitation, we used ROC curves to identify the best cut-off points for each of the extracranial carotid doppler parameters which best predicted improved functional outcome. These were further validated by comparing the ORs for improved outcome between groups using values that were either within or outside the cutoff point for each parameter. The PPVs of the studied PI and RI were all above or equal to 80%. This result denotes our study has obtained high accuracy in terms of prediction in stroke patients’ long term outcomes after thrombectomy surgery.

As shown in Table 4, an ipsilateral VA PI value of ≤2.3 meant the likelihood for significant improvement in the NIHSS score after 1 year of the procedure was 10 times higher than the group with ipsilateral VA PI >2.3 (OR = 0.102, *P*-value = .008). Similarly, the patient was 3 times more likely to have an improved mRS score when the ipsilateral VA PI values was >1.92 (OR = 0.337, *P*-value = .034) and 3.5 times more likely to have an improved mRS score when the ipsilateral ICA RI value was >0.75 (OR = 0.287, *P*-value = .021), compared with values outside the cutoff points that we identified. Interestingly, when the contralateral ICA RI value was ≤0.7, we observed that patients were more than 3.5 times likely to have improved Barthel index 1 year after the treatment (contralateral ICA RI >0.7: OR = 0.288, *P*-value = .020).

Most of the studied population (88%) revealed fair improvement in NIHSS score 1 year after ITT followed by IAT. However, through the results of ROC curve and multivariate regression analyses, the data elucidates that there seems to be a significant correlation between stenosis of ICA and/or VA and improvement in mRS and/or Barthel index. Once recanalization treatment ITT fails, or ipsilateral ICA PI/RI and VA RI are high, mRS score is difficult to improve; likewise, once ipsilateral VA RI and plaque index are too high, or even the contralateral ICA is stenosed, Barthel index is difficult to improve (Tables 3 and 4). As mentioned above, the detection of PI and RI values is important, since it could potentially be used to predict patients’ long term functional outcomes.

A major strength of this study is low heterogeneity among participants, given that most patients came from the surrounding local communities and shared the same ethnic group. Other strengths were technical consistency, as the same technician performed all the carotid duplex scans, and the ability to compare each patient's functional scores 1 year after the treatment allowed adequate time to also observe functional improvement. However, this could also potentially introduce confounding factors, such as different types of rehabilitation programs that participants may have attended within the year. The major shortcoming of the present study is of relatively small sample size (N = 92) as well as the fact there was no placebo group for comparison. However, the identification of new parameters which have the potential to predict when a patient is up to 10 times more likely to have an improved outcome is promising and warrants further investigation.

## 5 Conclusion

Carotid duplex scanning is a valuable, noninvasive, and portable tool which may assist in predicting the functional outcomes of AIS patients who have undergone ITT and IAT procedures. Both PI and RI appeared to have correlation with treatment response; with RI appeared to have stronger predictive power in patients with anterior circulation (ICA and CCA) occlusion, while posterior circulation (VA) occlusion was better predicted by PI. Assessment of PI and RI could, therefore, provide critical information for making clinical decisions regarding ITT plus IAT procedures in individual patients.

## Acknowledgments

The authors would like to thank Stroke Center, Changhua Christian Hospital for the technical support; Wen-Pei Wu, MD PHD for helping in IRB preparation and critical paper appraisal; Shih-Chun Wang, BS for data collection and initial data analysis

## Author contributions

**Formal analysis:** Chih-Ming Lin, Yu-Jun Chang.

**Methodology:** Chi-Kuang Liu, Chih-Ming Lin.

**Validation:** Shih-Liang Chang.

**Writing – original draft:** Chih-Ming Lin, Yang-Hao Ou, Yu-Jun Chang.

**Writing – review and editing:** Wei-Liang Chen, Yu-Jun Chang.

## Supplementary Material

Supplemental Digital Content
