# Supplementary material for: Carotid duplex parameters to predict long term outcomes of ischemic stroke patients receiving intra-arterial thrombectomy treatment
Source: Medicine (Baltimore). 2019 May 17;98(20):e15734. doi: 10.1097/MD.0000000000015734 (PMC6531233; doi:10.1097/MD.0000000000015734)
Supplement: Supplemental Digital Content [file medi-98-e15734-s001.doc]

**Table 1**

Correlation between various predictors.

|  | (n=92) | CT ASPECTS-admission | Ischemic core | Mismatch | Perfusion Tmax | NIHSS-admission | mRS-admission | Barthel index-admission | Plaque index-ipsilateral | Plaque index-contralateral | Duplex plaque type | IMT-ipsilateral |
| --- | --- | --- | --- | --- | --- | --- | --- | --- | --- | --- | --- | --- |
| CT ASPECTS-admission | r | 1.000 | -0.048 | -0.004 | 0.069 | 0.092 | -0.046 | 0.077 | -0.165 | -0.027 | -0.187 | 0.012 |
|  | p-value |  | 0.647 | 0.972 | 0.512 | 0.382 | 0.662 | 0.463 | 0.117 | 0.798 | 0.074 | 0.906 |
|  |  |  |  |  |  |  |  |  |  |  |  |  |
| Ischemic core | r | -0.048 | 1.000 | -0.287 | 0.448 | 0.120 | 0.060 | -0.113 | -0.088 | -0.007 | -0.089 | 0.146 |
|  | p-value | 0.647 |  | 0.006 | <0.001 | 0.255 | 0.570 | 0.285 | 0.407 | 0.946 | 0.397 | 0.165 |
|  |  |  |  |  |  |  |  |  |  |  |  |  |
| Mismatch | r | -0.004 | -0.287 | 1.000 | 0.190 | 0.019 | 0.056 | -0.119 | 0.064 | -0.046 | -0.006 | -0.012 |
|  | p-value | 0.972 | 0.006 |  | 0.069 | 0.861 | 0.595 | 0.260 | 0.541 | 0.666 | 0.955 | 0.913 |
|  |  |  |  |  |  |  |  |  |  |  |  |  |
| Perfusion Tmax | r | 0.069 | 0.448 | 0.190 | 1.000 | 0.118 | 0.072 | -0.116 | 0.083 | 0.050 | -0.213 | 0.206 |
|  | p-value | 0.512 | <0.001 | 0.069 |  | 0.261 | 0.495 | 0.271 | 0.433 | 0.636 | 0.041 | 0.049 |
|  |  |  |  |  |  |  |  |  |  |  |  |  |
| NIHSS-admission | r | 0.092 | 0.120 | 0.019 | 0.118 | 1.000 | 0.447 | -0.390 | 0.168 | 0.087 | 0.049 | 0.074 |
|  | p-value | 0.382 | 0.255 | 0.861 | 0.261 |  | <0.001 | <0.001 | 0.110 | 0.410 | 0.640 | 0.484 |
|  |  |  |  |  |  |  |  |  |  |  |  |  |
| mRS-admission | r | -0.046 | 0.060 | 0.056 | 0.072 | 0.447 | 1.000 | -0.683 | 0.113 | 0.035 | -0.013 | -0.065 |
|  | p-value | 0.662 | 0.570 | 0.595 | 0.495 | <0.001 |  | <0.001 | 0.281 | 0.741 | 0.903 | 0.541 |
|  |  |  |  |  |  |  |  |  |  |  |  |  |
| Barthel index-admission | r | 0.077 | -0.113 | -0.119 | -0.116 | -0.390 | -0.683 | 1.000 | -0.104 | -0.043 | 0.154 | 0.036 |
|  | p-value | 0.463 | 0.285 | 0.260 | 0.271 | <0.001 | <0.001 |  | 0.324 | 0.684 | 0.142 | 0.730 |
|  |  |  |  |  |  |  |  |  |  |  |  |  |
| Plaque index-ipsilateral | r | -0.165 | -0.088 | 0.064 | 0.083 | 0.168 | 0.113 | -0.104 | 1.000 | 0.672 | 0.083 | 0.434 |
|  | p-value | 0.117 | 0.407 | 0.541 | 0.433 | 0.110 | 0.281 | 0.324 |  | <0.001 | 0.430 | <0.001 |
|  |  |  |  |  |  |  |  |  |  |  |  |  |
| Plaque index-contralateral | r | -0.027 | -0.007 | -0.046 | 0.050 | 0.087 | 0.035 | -0.043 | 0.672 | 1.000 | 0.158 | 0.352 |
|  | p-value | 0.798 | 0.946 | 0.666 | 0.636 | 0.410 | 0.741 | 0.684 | <0.001 |  | 0.134 | 0.001 |
|  |  |  |  |  |  |  |  |  |  |  |  |  |
| Plaque type-carotid duplex | r | -0.187 | -0.089 | -0.006 | -0.213 | 0.049 | -0.013 | 0.154 | 0.083 | 0.158 | 1.000 | 0.087 |
|  | p-value | 0.074 | 0.397 | 0.955 | 0.041 | 0.640 | 0.903 | 0.142 | 0.430 | 0.134 |  | 0.409 |
|  |  |  |  |  |  |  |  |  |  |  |  |  |
| IMT-ipsilateral | r | 0.012 | 0.146 | -0.012 | 0.206 | 0.074 | -0.065 | 0.036 | 0.434 | 0.352 | 0.087 | 1.000 |
|  | p-value | 0.906 | 0.165 | 0.913 | 0.049 | 0.484 | 0.541 | 0.730 | <0.001 | 0.001 | 0.409 |  |
| ASPECTS: The Alberta Stroke Program Early CT Score; NIHSS: National Institue of Health Stroke Scale; mRS: modified Rankin Scale; IMT: intima media thickness; r: Pearson correlation coefficient. | | | | | | | | | | | | |

**Table 2**

Correlation between PI and RI of each artery.

| **CCA** | (n=92) | RI-ipsilateral | PI-ipsilateral | RI-contralateral | PI-contralateral |  | **ICA** | (n=92) | RI-ipsilateral | PI-ipsilateral | RI-contralateral | PI-contralateral |
| --- | --- | --- | --- | --- | --- | --- | --- | --- | --- | --- | --- | --- |
| RI-ipsilateral | r | 1.000 | 0.513 | 0.429 | 0.364 |  | RI-ipsilateral | r | 1.000 | 0.449 | 0.486 | 0.415 |
|  | p-value |  | <0.001 | <0.001 | <0.001 |  |  | p-value |  | <0.001 | <0.001 | <0.001 |
|  |  |  |  |  |  |  |  |  |  |  |  |  |
| PI-ipsilateral | r | 0.513 | 1.000 | 0.291 | 0.535 |  | PI-ipsilateral | r | 0.449 | 1.000 | 0.232 | 0.323 |
|  | p-value | <0.001 |  | 0.005 | <0.001 |  |  | p-value | <0.001 |  | 0.026 | 0.002 |
|  |  |  |  |  |  |  |  |  |  |  |  |  |
| RI-contralateral | r | 0.429 | 0.291 | 1.000 | 0.669 |  | RI-contralateral | r | 0.486 | 0.232 | 1.000 | 0.628 |
|  | p-value | <0.001 | 0.005 |  | <0.001 |  |  | p-value | <0.001 | 0.026 |  | <0.001 |
|  |  |  |  |  |  |  |  |  |  |  |  |  |
| PI-contralateral | r | 0.364 | 0.535 | 0.669 | 1.000 |  | PI-contralateral | r | 0.415 | 0.323 | 0.628 | 1.000 |
|  | p-value | <0.001 | <0.001 | <0.001 |  |  |  | p-value | <0.001 | 0.002 | <0.001 |  |
|  |  |  |  |  |  |  |  |  |  |  |  |  |
|  |  |  |  |  |  |  |  |  |  |  |  |  |
| **ECA** | (n=92) | RI-ipsilateral | PI-ipsilateral | RI-contralateral | PI-contralateral |  | **VA** | (n=92) | RI-ipsilateral | PI-ipsilateral | RI-contralateral | PI-contralateral |
| RI-ipsilateral | r | 1.000 | -0.129 | 0.012 | 0.161 |  | RI-ipsilateral | r | 1.000 | 0.510 | 0.341 | 0.338 |
|  | p-value |  | 0.220 | 0.910 | 0.125 |  |  | p-value |  | <0.001 | 0.001 | 0.001 |
|  |  |  |  |  |  |  |  |  |  |  |  |  |
| PI-ipsilateral | r | -0.129 | 1.000 | 0.071 | 0.138 |  | PI-ipsilateral | r | 0.510 | 1.000 | 0.124 | 0.237 |
|  | p-value | 0.220 |  | 0.500 | 0.189 |  |  | p-value | <0.001 |  | 0.240 | 0.023 |
|  |  |  |  |  |  |  |  |  |  |  |  |  |
| RI-contralateral | r | 0.012 | 0.071 | 1.000 | -0.171 |  | RI-contralateral | r | 0.341 | 0.124 | 1.000 | 0.317 |
|  | p-value | 0.910 | 0.500 |  | 0.103 |  |  | p-value | 0.001 | 0.240 |  | 0.002 |
|  |  |  |  |  |  |  |  |  |  |  |  |  |
| PI-contralateral | r | 0.161 | 0.138 | -0.171 | 1.000 |  | PI-contralateral | r | 0.338 | 0.237 | 0.317 | 1.000 |
|  | p-value | 0.125 | 0.189 | 0.103 |  |  |  | p-value | 0.001 | 0.023 | 0.002 |  |
| CCA: common carotid artery; ICA: internal carotid artery; ECA: external carotid artery; VA: vertebral artery; PI: pulsatility index; RI: resistance index; r: Pearson correlation coefficient. | | | | | | | | | | | | |
